# CD34^+^THY1^+^ synovial fibroblast subset in arthritic joints has high osteoblastic and chondrogenic potentials in vitro

**DOI:** 10.1186/s13075-022-02736-7

**Published:** 2022-02-15

**Authors:** Seiji Noda, Tadashi Hosoya, Yoji Komiya, Yasuhiro Tagawa, Kentaro Endo, Keiichiro Komori, Hideyuki Koga, Yasuhiro Takahara, Kazutaka Sugimoto, Ichiro Sekiya, Tetsuya Saito, Fumitaka Mizoguchi, Shinsuke Yasuda

**Affiliations:** 1 Department of Rheumatology, Graduate School of Medical and Dental Sciences, Tokyo Medical and Dental University, 1-5-45, Yushima, Bunkyo-ku, Tokyo, 113-8519 Japan; 2 Center for Stem Cell and Regenerative Medicine, Tokyo Medical and Dental University, Tokyo, Japan; 3 Department of Joint Surgery and Sports Medicine, Graduate School of Medical and Dental Sciences, Tokyo Medical and Dental University, Tokyo, Japan; 4 Department of Orthopedics, Nippon Koukan Fukuyama Hospital, Fukuyama, Japan; 5 Department of Orthopedics, Sonodakai Joint Replacement Center Hospital, Tokyo, Japan

## Abstract

**Objective:**

Synovial fibroblasts (SFs) in rheumatoid arthritis (RA) and osteoarthritis (OA) play biphasic roles in joint destruction and regeneration of bone/cartilage as mesenchymal stem cells (MSCs). Although MSCs contribute to joint homeostasis, such function is impaired in arthritic joints. We have identified functionally distinct three SF subsets characterized by the expression of CD34 and THY1 as follows: CD34^+^THY1^+^, CD34^−^THY1^−^, and CD34^−^THY1^+^. The objective of this study was to clarify the differentiation potentials as MSCs in each SF subset since both molecules would be associated with the MSC function.

**Methods:**

SF subsets were isolated from synovial tissues of 70 patients (RA: 18, OA: 52). Expressions of surface markers associated with MSCs (THY1, CD34, CD73, CD271, CD54, CD44, and CD29) were evaluated in fleshly isolated SF subsets by flow cytometry. The differentiation potentials of osteogenesis, chondrogenesis, and adipogenesis were evaluated with histological staining and a quantitative polymerase chain reaction of differentiation marker genes. Small interfering RNA was examined to deplete THY1 in SFs.

**Results:**

The expression levels of THY1^+^, CD73^+^, and CD271^+^ were highest and those of CD54^+^ and CD29^+^ were lowest in CD34^+^THY1^+^ among three subsets. Comparing three subsets, the calcified area, alkaline phosphatase (ALP)-stained area, and cartilage matrix subset were the largest in the CD34^+^THY1^+^ subset. Consistently, the expressions of differentiation markers of the osteoblasts (*RUNX2*, *ALPL*, and *OCN*) or chondrocytes (*ACAN*) were the highest in the CD34^+^THY1^+^ subset, indicating that the CD34^+^THY1^+^ subset possessed the highest osteogenic and chondrogenic potential among three subsets, while the differentiation potentials to adipocytes were comparable among the subsets regarding lipid droplet formations and the expression of *LPL* and *PPARγ*. The knockdown of THY1 in bulk SFs resulted in impaired osteoblast differentiation indicating some functional aspects in this stem-cell marker.

**Conclusion:**

The CD34^+^THY1^+^ SF subset has high osteogenic and chondrogenic potentials. The preferential enhancement of MSC functions in the CD34^+^THY1^+^ subset may provide a new treatment strategy for regenerating damaged bone/cartilage in arthritic joints.

**Supplementary Information:**

The online version contains supplementary material available at 10.1186/s13075-022-02736-7.

## Background

In rheumatoid arthritis (RA) and osteoarthritis (OA), the joint function is impaired due to cartilage and bone damage. The affected patients suffered from the decline of physical status, resulting in a shortening in healthy life expectancy [1]. The homeostatic responses of the joint environments, including the joint repairing, were impaired because of chronic inflammation and mechanical stress [2]. According to these clinical situations, the strategy to enhance tissue regeneration would be a possible strategy to construct complementary treatment.

Synovial fibroblasts (SFs) are contributing to the pathogenesis of RA and OA by secreting inflammatory cytokines, tissue degrading factors, and making pannus formation [2, 3]. SFs also possess several characteristics of mesenchymal stem cells (MSCs), including self-renewal capacity and multi-lineage differentiation potentials to mesenchymal tissues [4, 5]. MSCs isolated from synovial tissue can be more efficiently differentiated into chondrocytes than those from other tissues (e.g., bone marrow) [6, 7]. In addition, MSCs are proliferated in response to mechanical stress or inflammatory cytokines [8, 9], suggesting that they can be induced under disease conditions in RA and OA. Since MSC functions as osteogenesis and chondrogenesis were induced in case of bone damages or bone fractures [10–12], the therapeutic application of synovial MSCs would have a potent for the repairment of bone erosion in RA.

We previously reported that SFs in arthritic joints are composed of three functionally distinct subsets, CD34^−^THY1^−^, CD34^−^THY1^+^, and CD34^+^THY1^+^ population, based on the expression of CD34 and THY1 [13]. The CD34^−^THY1^+^ subset was found to be expanded in patients with RA, whereas the proportion of the CD34^+^THY1^+^ subset was comparable between RA and OA. Both subsets possessed pathological functions, including proinflammatory cytokine secretions, high proliferation capacity, and enhanced invasiveness when compared with the CD34^−^THY1^−^ subset. THY1 and CD34 are the MSC surface markers related to wound repair as well as lineage potentials in osteogenesis and chondrogenesis [14, 15]. Since we and others have already elucidated that the functions of THY1^−^/THY1^+^ SF are distinct [16–18], we hypothesized that three SF subsets have a different function as MSC and that baseline expression of THY1 in SF subsets would determine the differentiation potentials. Indeed, the THY1^+^CD73^+^ SF subset presented higher chondrogenic potentials than the THY1^−^CD73^+^ subset [19].

In the present study, we evaluated the MSC function of each SF subset regarding differentiation potential to osteoblast, chondrocyte, and adipocyte. Here we demonstrate that the CD34^+^THY1^+^ subset has MSC potential than the others regardless of the background diseases, suggesting future therapeutic applications utilizing MSC function in this subset.

## Methods

### Patient recruitment and isolation of synovial cells

We obtained synovial tissues from surgeries of joint replacement for patients with OA and RA. We consecutively collected all the available OA and RA samples for this study. Written informed consent for this study and the ethics approval from the medical research ethics committee of Tokyo Medical and Dental University (approval number: M2000-979) were obtained. The synovial tissues were collected from 70 patients (RA: 18, OA: 52). All the patients with RA have been received treatment with disease-modified anti-rheumatic drugs (DMARDs) including biologics. Ten patients were administered steroids (average dose, 2.9 mg/day). Other characteristics are described in Table 1. In this study, we statistically analyzed combined data from RA and OA samples because of their limited sample numbers. Our previous study demonstrated that the altered proportions of SF subsets but not subsets themselves differed between RA and OA [13].

**Table 1:** Patient characteristics

	RA	OA
Number of patients	18	52
Treatment	bDMARDs: 7	ND
	cDMARDs: 14	ND
	(MTX: 10, others: 6)	ND
Average dose of prednisolone (mg/day)	2.9 (1–10)	ND
Average C reactive protein (mg/dL)	1.20 (0.02–6.04)	ND
Proportion of SF subsets		
CD34^−^THY1^−^	35.5% (8.1–83.2)	56.6% (22.1–87.1)
CD34^−^THY1^+^	24.1% (1.6–64.2)	10.2% (1.0–30.9)
CD34^+^THY1^+^	24.8% (2.1–55.7)	17.2% (0.8–56.9)

*bDMARDs* biologic disease-modified anti-rheumatic drugs, *cDMARDs* conventional disease-modified anti-rheumatic drugs, *MTX* methotrexate

Tissue samples were collected consecutively from joint replacement surgeries to eliminate any bias as previously reported [13]. Briefly, joint tissues were obtained immediately after the surgeries, followed by removal of bone and adipose tissues with scissors. Synovial tissues were minced into small pieces and then subjected to enzymatic digestion. For cell culture, we digested tissues with 2 mg/mL collagenase type 4 (Worthington, NJ, USA), 0.8 mg/mL Dispase II, and 0.1 mg/mL DNase I (Roche, Basel, Switzerland) in Dulbecco’s modified Eagle Roche’s medium (DMEM) at 37 °C. After 15 min, we collected the supernatant and replaced it with a fresh enzyme mix. These procedures were repeated every 15 min for a total of 1 h. After lysing red blood cells with ACK-lysing buffer, obtained cells were treated with antibodies as described below then sorted by FACS Aria II and III (BD Biosciences, CA, USA) with 100-μm nozzle.

### Antibodies

The following antibodies and reagents were used for the analysis of synovial cells with flow cytometry and cell sorting: anti-CD45-APC-H7 (2D1, BD Biosciences, CA, USA), anti-CD235a-APC-Alexa Fluor750 (11E4B-7-6, Beckman Coulter, FL, USA), anti-CD31-PE-Cyanine7 (WM-59, eBioscience, CA, USA), anti-CD146-APC (P1H12, eBioscience), anti-CD34-PE (4H11, eBioscience), anti-PDPN-PerCP-eFluor710 (NZ-1.3, eBioscience), anti-THY1-FITC (5E10, BD Bioscience), anti-CD73-PE-CF594 (AD2, BD Bioscience), anti-CD271-APC (ME20.4, eBioscience), anti-CD54-PE-CF594 (HA58 BioLegend, CA, USA), anti-CD44-APC (G44-26 BD Bioscience), anti-CD29-APC (TS2/16 BioLegend), human TruStain FcX (BioLegend), and Live/Dead fixable aqua dead cell stain kits (Molecular Probes, Thermo Fisher Scientific, MA, USA).

### Flow cytometry analysis

The gating strategy of SF subsets was as shown (Supplementary Figure 1). While the mean ratios of CD34^−^THY1^−^, CD34^−^THY1^+^, and CD34^+^THY1^+^ subsets in RA were 35.5%, 24.1%, and 24.8%, respectively, the mean ratios of these subsets in OA were 56.6%, 10.2%, and 17.2%. These results were compatible as previously reported [13]. The mean purity of each subset after sorting was 95.9%, 94.3%, and 98.0%, respectively.

We evaluated the expression of MSC surface markers (THY1, CD34, CD73, CD271, CD54, CD29, and CD44) in the SF subsets. Considering the expression of MSC markers in the freshly isolated synovial cells, we evaluated the mean fluorescence intensity (MFI) as expression levels of these markers in the individual subsets by flow cytometry in the 12 consecutive samples (RA: 3, OA: 9) (Supplementary Figure 2).

### Cell culture

We sorted CD34^−^THY1^−^ fibroblasts, CD34^−^THY1^+^ fibroblasts, and CD34^+^THY1^+^ fibroblasts and cultured them in DMEM supplemented with 10% FBS (Gemini Bio, CA, USA), 2 mM l-glutamine, antibiotics (penicillin and streptomycin), and essential and nonessential amino acids (Life Technologies, CA, USA). The cells were expanded for 20–30 days for assays.

### Osteoblast, chondrogenic, and adipocyte differentiation

Osteoblastic induction was performed as previously reported [6]. 3.0 × 10^3^ cells/cm^2^ were plated in a 12-well plate in an osteogenic differentiation medium containing l-ascorbic acid-2-phosphate (0.2 mM; Wako Pure Chemical Industries, Osaka, Japan), beta-glycerophosphate (5 mM; Wako Pure Chemical Industries), and dexamethasone (1 nM; Wako Pure Chemical Industries) and incubated at 37 °C in 5% CO_2_. All media were changed twice per week. Each SF subset was cultured for 3–4 weeks. Histological staining was performed with alizarin red (Merk Millipore, MA, USA) and alkaline phosphatase (ALP) staining for osteoblast differentiation.

For chondrogenic differentiation, 1.25–2.5 × 10^5^ cells were placed in a 15-mL polypropylene tube (AGC Techno Glass Co., Ltd, Shizuoka, Japan) and centrifuged at 1500 × *g* for 5 min. The cells were cultured in a chondrogenic induction medium containing 1000 ng/mL of BMP-2 (PeproTech, NJ, USA) and 10 ng/mL of transforming growth factor-β3 (PeproTech), incubated at 37 °C in 5% CO_2_ for 3 weeks. All media were changed twice per week. Histological staining was performed with safranin O staining for chondrogenesis.

For adipogenesis, 7.0 × 10^3^ cells/cm^2^ are plated and cultured in StemPro™ Adipogenesis Differentiation Kit (Gibco, Thermo Fisher Scientific, MA, USA) for 3 weeks. All media were changed twice per week. Oil-red staining was used to evaluate adipogenesis.

For chondrogenesis and adipocyte differentiation, we referred to the previous reports with some modifications [20, 21]. Briefly, we performed some pellet culture at a density of 1.25 × 10^5^ cells due to an imbalance in the number of each subset.

### Quantitative real-time polymerase chain reaction (qPCR)

cDNA was synthesized with QuantiTect Reverse Transcription kit (Qiagen, Hilden, German). Quantitative polymerase chain reaction (qPCR) was performed with Brilliant III Ultra-Fast SYBR Green qPCR master mix (Agilent Technologies, CA, USA) on a LightCycler96® (Roche). The following primers are used as shown in Table 2.

**Table 2:** Primer sequences used in the study

Primer	Forward	Reverse
ALPL	5′ATGCTGAGTGACACAGACAAGAAG	5′GGTAGTTGTTGTGAGCATAGTCCAC
RUNX2	5′CATCACCGATGTGCCTAGG	5′TAAGTAAAGGTGGCTGGATAGTG
OCN	5′ GACTGTGACGAGTTGGCTG	5′ GGGAAGAGGAAAGAAGGGTG
ACAN	5′ TGTGGGACTGAAGTTCTTGG	5′AGCGAGTTGTCATGGTCTG
LPL	5′ACACTTGCCACCTCATTCC	5′ ACCCAACTCTCATACATTCCTG
PPARγ	5′GTCGGTTTCAGAAATCGGTTG	5′ GCTGGTCGATATCACTGGAG
THY1	5′ CTACTTATCCGCCTTCACTAGC	5′ TGATGCCCTCACACTTGAC
CD34	5′ CAACATCTCCCACTAAACCCT	5′ TCTTAAACTCCGCACAGCTG
CD73	5′ CACACGGATGAAATGTTCTGG	5′ GGTCAAATGTGCCTCCAAAG
CD271	5′ GTGGAGAGTCTGTGCAGTG	5′ ATCGGTTGTCGGAATGTGG
CD54	5′ CAATGTGCTATTCAAACTGCCC	5′ CAGCGTAGGGTAAGGTTCTTG
CD29	5′ CAATGTGCTATTCAAACTGCCC	5′CAGCGTAGGGTAAGGTTCTTG
18S	5′ACTCAACACGGGAAACCTCA	5′AACCAGACAAATCGCTCCAC

*ALPL* alkaline phosphatase, *OCN* osteocalcin, *ACAN* aggrecan, *LPL* lipoprotein lipase, *PPARγ* peroxisome proliferator-activated receptor γ

### Knockdown of gene expression by small interfering (si) RNA

Bulk SFs were seeded at 1.2 × 10^4^ into 12-well cell culture plates and subsequently transiently transfected with 20 pM of THY1 or control small interfering (si) RNA (Thermo Fisher Scientific) using Lipofectamine RNAiMax (Thermo Fisher Scientific) according to the manufacturer’s protocol. Cells were incubated with siRNA for 3 days and subjected to osteogenic differentiation as described above.

### Cell survival/proliferation assay

Cell survival/proliferation was evaluated with a water-soluble tetrazolium salt (WST-8) colorimetric assay using Cell Counting Kit-8 (Dojindo) according to the manufacturer’s protocol. Briefly, SFs transfected with siRNAs were incubated with 1% WST-8 for 6 h and absorbance of supernatants at 450 nm was measured by a plate reader.

## Results

### CD34^+^THY1^+^ subset expressed MSC surface markers

**Fig. 1:**
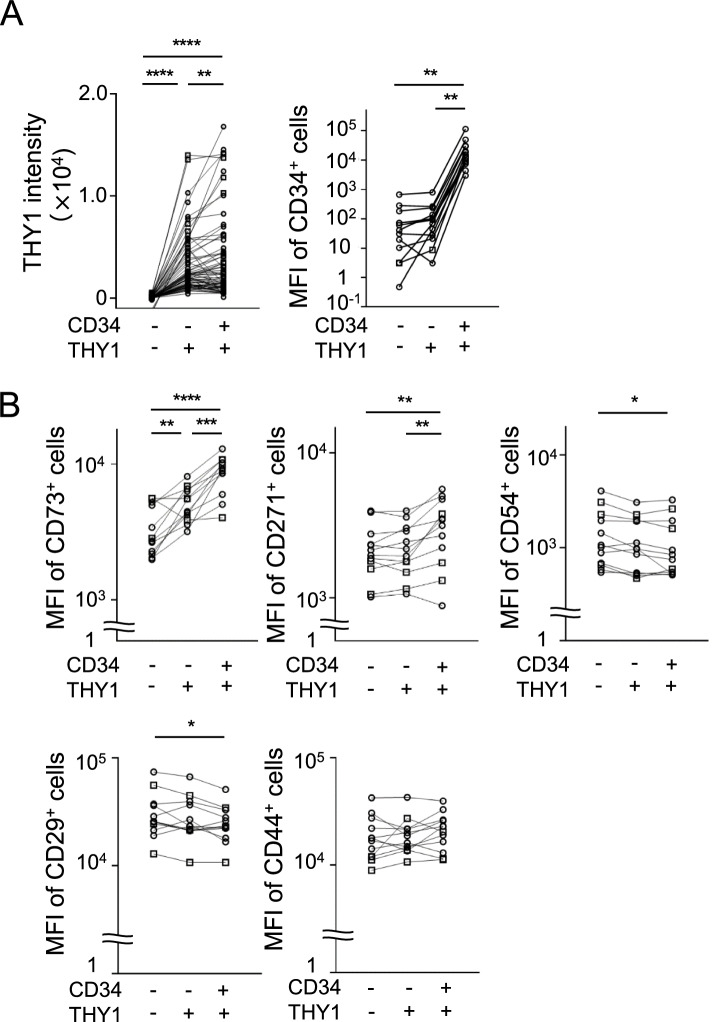
The expression of THY1, CD34, and MSC surface markers in the individual subset. **A** THY1 and CD34 expression. The mean fluorescence intensity (MFI) of THY1 and CD34 was evaluated with flow cytometry. Data was shown in total samples (OA+RA). **B**–**D** MSC surface markers. The MFI of MSC surface markers (CD73, CD271, CD54, CD29, and CD44) was evaluated with flow cytometry. OA and RA samples are plotted as white circles and white squares, respectively. Data were analyzed by Holm-Sidak’s multiple comparison test for comparing each subset from the same samples (*p < 0.05, **p < 0.01, ***p < 0.001, ****p < 0.0001)

Considering the expression of surface markers in the freshly isolated synovial cells, both CD34^+^THY1^+^ and CD34^−^THY1^+^ subsets expressed THY1 with higher levels than the CD34^−^THY1^−^ subset, as we have shown previously [13]. Interestingly, the expression level of THY1 in CD34^+^THY1^+^ was the highest among the SF subsets regardless of the underlying diseases (Fig. 1A).

The expressions of CD73 and CD271 were the highest in the CD34^+^THY1^+^ subset among three subsets (Fig. 1B), whereas the expression levels of CD54 and CD29, a surface marker with low osteogenic potentials derived from other cell types [22, 23], were lower in the CD34^+^THY1^+^ subset than the CD34^−^THY1^−^ subset (Fig. 1B). The expression level of CD44 was not significantly different among the three subsets (Fig. 1B). We also analyzed data from RA and OA separately (Supplementary Figure 6). Although statistical significance was observed in only RA + OA and OA data, RA and OA showed the same tendency.

Regarding the transcriptions of MSC surface markers in microarray data, these were not significantly different among the three subsets (Supplementary Figure 3), indicating that these molecules are not regulated differently at the transcriptional levels. We also evaluated the expression level of surface THY1, CD34, and CD73 by flow cytometry after 4-week expansion for the subsequent differentiation experiments (Supplementary Figure 4). The expression of CD34 in the CD34^+^THY1^−^ subset was lost after expansion, while that of THY1 and CD73 was comparable among the three subsets.

### CD34^+^ THY1^+^ subset presented the highest osteogenic potentials

**Fig. 2:**
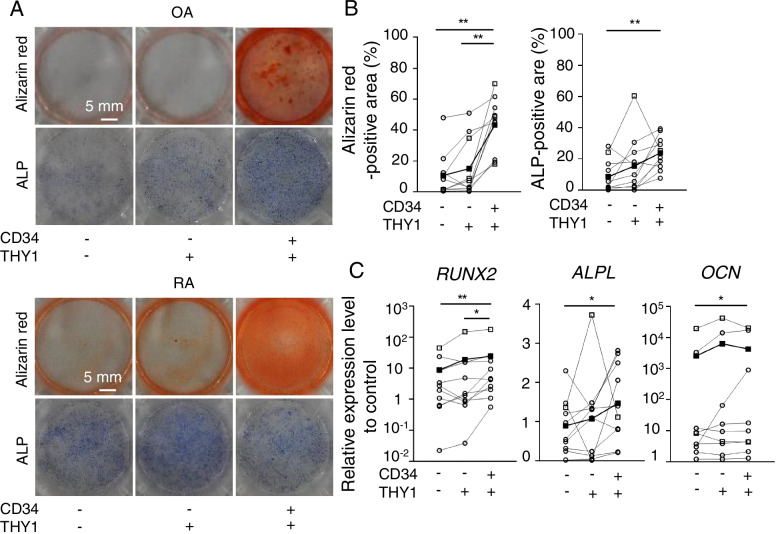
Osteogenic potentials of SF subsets. **A** Representative photographs of whole dishes stained with alizarin red and ALP. Individual subsets were incubated in the calcification differentiation medium for 3 weeks. **B** Quantification of the staining. Cells per donor were cultured in 3 dishes and the results of 10 donors for alizarin red and 11 donors for ALP were plotted. **C** Gene expression levels by qPCR. The expression levels of the genes were plotted as a ratio to the expression level of 18S of undifferentiated SF as a control. Cells per donor were cultured in 3 dishes and the results of 10 donors for *RUNX2* and *ALPL* and 9 donors for *OCN*. OA and RA samples are plotted as white circles and white squares, respectively. The average data of each subset are plotted as black squares. Data were analyzed by Dunn’s multiple comparison test for comparing each subset from the same patients (*p < 0.05, **p < 0.01)

The average ratio of calcified area stained with alizarin red as red spots was 4 and 1.5 times higher in CD34^+^THY1^+^ subsets than CD34^−^THY1^−^ and CD34^−^THY1^+^ subsets (Fig. 2A, B). We also stained three SF subsets with ALP staining to verify whether observed calcification is due to the differentiation of CD34^+^THY1^+^ SFs into osteoblasts. The activity of ALP, which was expressed as blue-stained area, was observed at twice higher levels in the CD34^+^ THY1^+^ subset (Fig. 2A, B) than the CD34^−^THY1^−^ subset.

To confirm the osteoblast differentiation by quantifying differentiation-associated genes, the expressions of mRNA levels were measured. The expression levels of *RUNX2*, the master regulator for osteoblast differentiation [24], were 2.8 times higher in the CD34^+^THY1^+^ subset than the CD34^−^THY1^−^ subset (Fig. 2C). As for expression levels of *ALPL*, which is a marker in the early stage of osteoblast differentiation, it was expressed 1.8 times higher in the CD34^+^THY1^+^ subset than the other two subsets after the differentiation for 3 weeks (Fig. 2C). The expression levels of *OCN*, a differentiation marker in the mature osteoblast, were 1.7 times higher in the CD34^+^THY1^+^ subset than the CD34^−^THY1^−^ subset after the differentiation for 4 weeks (Fig. 2C). These findings indicate that THY1^+^ subsets, especially the CD34^+^THY1^+^ subset, are the subset with the most superior osteogenic potentials in vitro. We also analyzed data from RA and OA separately (Supplementary Figure 7). Although statistical significance was observed in only RA + OA and OA data, RA and OA showed the same tendency.

### The CD34^+^ THY1^+^ subset possessed the highest chondrocyte differentiation potentials

**Fig. 3:**
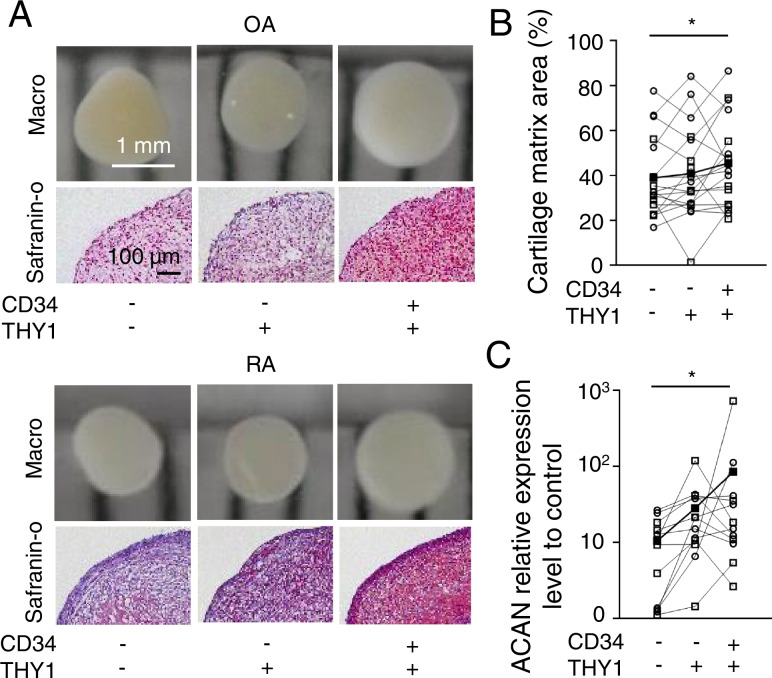
Chondrogenic potentials of SF subsets. **A**, **B** Size and histology of the chondrocyte pellets. Individual subsets were incubated in the chondrocyte differentiation medium for 3 weeks. Representative macro picture of chondrocyte pellet (**A**, upper). The chondrocyte matrix was stained as red by safranin O staining and measured the ratio of the stained area (**A**, lower **B**). Cells per donor were cultured in 3 pellets and the results of 18 donors (**C**). **C** qPCR of the chondrogenic differentiation marker. The relative expression levels of *ACAN* in differentiated cells. Cells per donor were cultured in 3 pellets and the results of 12 donors. The target genes were normalized with 18S of undifferentiated SF as a control. OA and RA samples are plotted as white circles and white squares, respectively. The average data of each subset are plotted as black squares. Data were analyzed by Dunn’s multiple comparison test for comparing each subset from the same samples (*p < 0.05)

After the culture in chondrogenic differentiation medium for 3 weeks, the largest chondrocyte pellets were formed from the CD34^+^THY1^+^ subset (Fig. 3A). The ratio of cartilage matrix stained as red by safranin O staining in CD34^−^THY1^−^, CD34^−^THY1^+^, and CD34^+^THY1^+^ subsets were 39%, 41%, and 46%, respectively (Fig. 3B). The CD34^+^THY1^+^ subset presented a significantly higher ratio of cartilage matrix than CD34^−^THY1^−^ subsets.

Confirming the quantitative verification of chondrogenesis, expression levels of *ACAN*, which codes aggrecan, were evaluated. In both CD34^−^THY1^+^ and CD34^+^THY1^+^ subsets, *ACAN* expression levels were 2.8 and 8 times higher than in the CD34^−^THY1^−^ subset, respectively (Fig. 3B). These findings indicated that THY1^+^ subsets, especially the CD34^+^THY1^+^ subset, have the highest chondrogenic potential in vitro. We also analyzed data from RA and OA separately (Supplementary Figure 8). Although statistical significance was observed in only RA + OA and OA data, RA and OA showed the same tendency.

### Adipocyte differentiation potential was not significantly different among the subsets

**Fig. 4:**
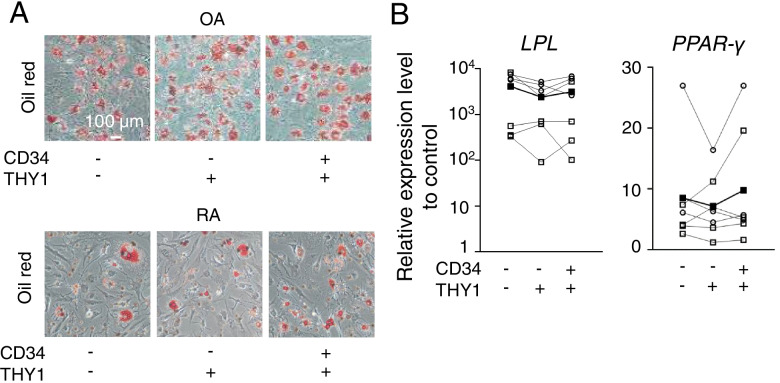
Adipogenic potential of SF subsets. **A** Oil-red staining. The SF subsets cultured in an adipocyte differentiation medium for 3 weeks. Adipogenesis was detected as red spots with oil-red staining. **B** qPCR of adipogenic differentiation markers. The relative expression levels of *LPL* and *PPARγ* in differentiated cells. Cells per donor were cultured in 3 dishes and the results of 7 donors. The target genes were normalized with 18S of undifferentiated SF as a control. OA and RA samples are plotted as white circles and white squares, respectively. The average data of each subset are plotted as black squares. Data were analyzed by Dunn’s multiple comparison test for comparing each subset from the same samples

After culture in the adipocyte differentiation medium for 3 weeks, all SF subsets comparably presented lipid droplets (Fig. 4A). The expressions of adipocyte-associated genes, including *LPL* and *PPARγ*, which codes lipoprotein lipase and peroxisome proliferator-activated receptor γ, were evaluated. The expression levels of *LPL* and *PPARγ* transcripts were not significantly different (Fig. 4B). We also analyzed data from RA and OA separately (Supplementary Figure 9). Although statistical significance was not observed, RA and OA showed the same tendency.

These findings indicated that osteogenic and chondrogenic potentials were relatively high in the CD34^+^THY1^+^ subset, whereas adipogenic potential was comparable among the three subsets.

### THY1 knockdown suppressed osteoblast differentiation

Since THY1 is one of the MSC surface markers and associated with lineage potentials in osteogenesis and chondrogenesis, we hypothesized that baseline expression of THY1 in SF subsets determined differentiation potentials. Therefore, we investigated the endogenous function of THY1 in the osteoblast differentiation by the depletion using RNA interference in bulk SFs.

THY1 knockdown had minor effects on the survival and proliferation of SFs as assessed by WST-8 assay (Supplementary Figure 5A). We also examined the effects of THY1 knockdown on the expression of other MSC markers (Supplementary Figure 5B). The expression of CD29 was significantly suppressed by THY1 knockdown. CD73 was also suppressed in most samples. We could not detect any changes in CD271 and CD54 expressions.

**Fig. 5:**
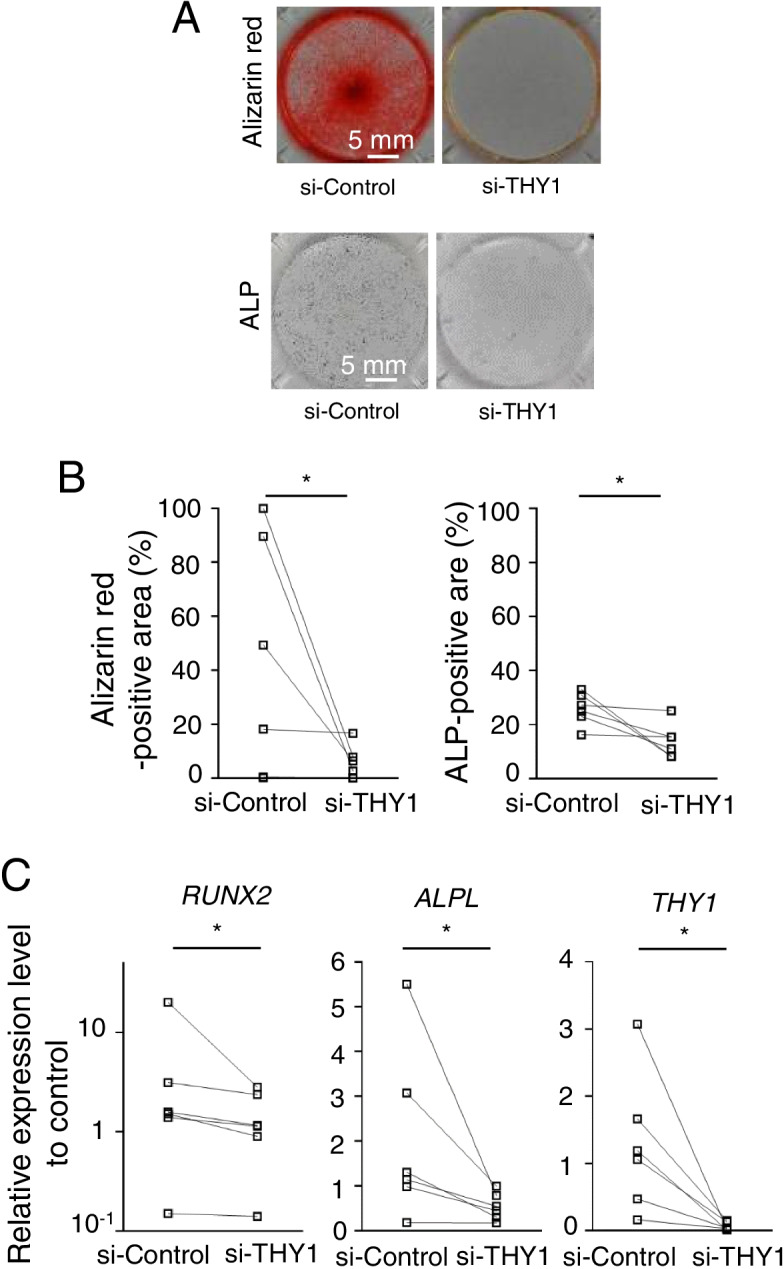
THY1 knockdown regulated osteoblast differentiation. **A** Alizarin red and ALP staining. Representative pictures of SFs stained by alizarin red and ALP staining after THY1 knockdown. **B** Quantification of staining. **C** qPCR of osteogenic differentiation markers. Comparison of osteoblast differentiation markers at day 7 after THY1 knockdown. Cells per donor were cultured in 2 dishes and the results of 6 donors. The target genes were normalized with 18S of undifferentiated SF as a control. Data were analyzed by Wilcoxon’s test for comparing each subset from the same samples (*p < 0.05)

Compared with those treated with siRNA control, THY1-deficient SF demonstrated impaired calcification as well as attenuated ALP activity after 4-week culture in an osteogenic differentiation medium (Fig. 5A, B). Expressions of *THY1*, *RUNX2*, and *ALPL* were also significantly suppressed (Fig. 5C). These findings supported the hypothesis that the expression of endogenous THY1 is inevitable for the MSC functions in the CD34^+^THY1^+^ subset.

## Discussion

In the present study, we identified significantly higher differentiation potency in the CD34^+^THY1^+^ subset by evaluating the osteogenic and chondrogenic differentiation potentials. The pattern of the MSC-associated surface markers also supported the highest potential as MSCs in the CD34^+^THY1^+^ subset.

Our findings were consistent with the previous reports that THY1 expression was associated with osteogenesis in bone marrow-derived MSCs [25]. Additionally, THY1 is involved in angiogenesis through the differentiation of endothelial cells [26]. In arthritic joints, perivascular SFs are exposed to Notch3 signaling from vascular endothelial cells, which is essential in the induction and maintenance of the expression of THY1 [27]. THY1^+^ subsets, which predominantly localize at the perivascular lesion in the synovium, may be induced due to angiogenesis followed by synovitis and joint damage. In contrast to the differentiation potentials, THY1 interacts with integrin and induces cell apoptosis via activation of the caspase 3/7 pathway [28, 29]. Since THY1 expression was enhanced in fibroblasts when joint tissue was injured [30, 31], these findings suggest that THY1 exerts to orchestrate the maintenance of joint homeostasis. In the arthritic joints, the CD34^+^THY1^+^ subset might be induced compensatory by the mechanical stress/injury in the joints.

Some MSC markers including THY1 are functionally involved in the lineage commitment. Among them, CD73, an ecto-5′-nucleotidase, which produces extracellular adenosine, affects the osteoblast differentiation of MSC [32]. It is interesting to note that CD73 is highly expressed in THY1^+^ subsets (Fig. 1A) and that THY1 knockdown suppressed CD73 expression. Thus, CD73 in addition to THY1 may be also responsible for the high osteogenic differentiation potential of THY1^+^ subsets. There is one study demonstrating opposing effects of THY1 on the osteogenic differentiation. They employed MSCs harvested from dental pulp, adipose tissue, and amniotic fluid, and the lentiviral shRNA transduction to archive THY1 knockdown [33]. These differences may explain the opposite results by them and others [34] including our study.

In addition to THY1, we identified CD34 as a complementary marker to enrich MSCs from freshly isolated synovial cells. Since CD34, a marker for hematopoietic stem cells [35], was used as a negative marker for MSC isolation so far, CD34 is positive in freshly isolated bone marrow-derived MSC (BMSC) and CD34^+^ BMSCs produced greater fibroblast colony-forming units than CD34^−^ BMSCs [36]. CD34 is also expressed not only on hematopoietic stem cells but also 10% of circulating fibrocytes, which are recruited to the injured site and associated with inflammation and wound repair [37–39]. Although the potentials to differentiate osteoblasts and chondrocytes circulating fibrocytes have been analyzed in fibrotic lung tissue [40], the differentiation potentials in SFs, especially CD34^+/−^ populations, have not been compared. Therefore, this is the first report evaluating the MSC function in the CD34^+^THY1^+^ double-positive SF subset.

In active arthritis, the osteogenic and chondrogenic differentiation potentials in THY1^+^ subsets may be overwhelmed or impaired due to inflammation. Although TNF and IL-6 blocking therapy have similar efficacy for RA patients [41], erosion repairment was observed most frequently under treatment with IL-6 blocking therapy [42, 43]. Since MSC functions were enhanced in the presence of inflammatory cytokines, including IL-1β or TNF α [8, 44], bone repair would result from the fine-tuning of inflammatory mediators in the arthritic joint. Suppression of several inflammatory cytokines ameliorates arthritis effectively, whereas these might not be beneficial for inducing MSC functions.

As the CD34^+^THY1^+^ subset has a highly potent MSC function, these cell populations might be appropriate to be applied for joint repairing therapy. However, the CD34^+^THY1^+^ subset would simultaneously contribute to the RA pathogenesis by the secretion of inflammatory cytokines and by enhanced proliferation potential [13]. We need to find the way to preferentially utilize the MSC function in THY1^+^ subsets or modify pro-inflammatory cytokine production in CD34^+^THY1^+^ for further therapeutic application. To enhance MSC potentials, we would consider several approaches to induce the expression of THY1^+^ in SFs. Jagged 1 and Delta-like 4, which are one of the Notch3 ligands, may be useful for THY1 induction [27, 45]. Stimulation of Notch3 signaling may be applicable to acquire osteogenic and chondrogenic differentiation potentials in SFs. A low oxygen environment is beneficial for MSC expansion and chondrogenesis by activating hypoxia-inducible factor α and upregulation of THY1 [46].

Direct introduction of MSCs in the inflammatory joints has been proved to have immunosuppressive effects by induction of inducible regulatory T cells, which leads to inhibition of T cell proliferation and cytokine production [47]. In addition, their systemic administration improves arthritis by inhibiting osteoclast differentiation in the arthritic animal model [48]. Clinical trials by administrating human MSCs into joint spaces have been examined [4, 49, 50]. Alternatively, MSC-derived exosomes, but not MSCs themselves, would be candidates for a novel treatment strategy for RA and OA since they can promote chondrogenesis [51].

Our study comprises several limitations. First, more than half of the synovial tissues were derived from OA, and the comparison of differentiation potential between RA and OA was not sufficient. Second, osteogenic and chondrogenic potentials in the CD34^+^THY1^+^ subset were not evaluated under the in vivo environment. Third, we had not analyzed the relevance of previously reported signaling pathways, including the Wnt pathway [52]. Fourth, we used bulk SFs for the THY1 knockdown experiment because of the limited cell number of each subset. Fifth, the functions of MSC surface markers other than THY1 were not examined.

## Conclusions

The multipotency of differentiation in SFs has drawn attention of researchers as such MSC-like characteristics would expect us to develop novel therapeutic strategies for joint repair in RA and/or OA. The CD34^+^THY1^+^ subset presented high osteogenic and chondrogenic differentiation potentials. This subset also most intensively expressed MSC-positive surface markers at the protein level, CD73 and CD271 as well as THY1. The preferential enhancement of MSC functions in the CD34^+^THY1^+^ subset may provide a new treatment strategy in the regeneration of damaged bone/cartilage in advanced RA and/or OA.

## Supplementary Information

**Additional file 1**: **Supplementary Figure 1**. Gating strategy. Gating strategy of freshly isolated cells from OA and RA synovial tissues using flow cytometry. Cells were stained with CD45, CD235a, CD31, THY1 and CD34. Individual subsets were analyzed for the expression of MSC surface markers and were sorted as CD34^-^THY1^-^, CD34^-^THY1^+^ and CD34^+^THY1^+^, respectively.

**Additional file 2**: **Supplementary Figure 2**. MFI measurement. Positive expression of THY1 and MSC surface markers in SF subsets was identified by comparing fluorescence-minus-one control. THY1 intensity was calculated as MFI of THY1^+^ SF population. Dash line indicated negative populations, while solid line indicated positive populations.

**Additional file 3**: **Supplementary Figure 3**. Expression of MSC surface markers. Expression levels of MSC surface markers (CD73, CD271, CD54, CD29 and CD44) on freshly isolated SF subsets were re-analyzed using microarray analysis previously reported (https://www.ncbi.nlm.nih.gov/geo/geo2r/?acc=GSE109450&platform=GPL18573).

**Additional file 4**: **Supplementary Figure 4**. Expression of MSC surface markers after expansion. The MFI of MSC surface markers (THY1, CD34 and CD73) was evaluated with flow cytometry. OA and RA samples are plotted as white circles and white squares, respectively. The average data of each subset are plotted as black squares. Data were analyzed by Holm-Sidak’s multiple comparison test for comparing each subset from the same samples (*p<0.05, **p<0.01).

**Additional file 5**: **Supplementary Figure 5**. Cell survival/proliferation and MSC surface marker expression by THY1 knockdown. WST-8 assay. Cell survival/proliferation by THY1 knockdown was assessed using WST-8 assay. Data were analyzed by Wilcoxon’s test for comparing each subset from the same samples (*p<0.05). MSC surface marker expression. Expression of MSC surface markers at day 7 after THY1 knockdown was examined by qPCR. The target genes were normalized with 18S of undifferentiated SF as a control. Data were analyzed by Wilcoxon’s test for comparing each subset from the same samples (*p<0.05).

**Additional file 6**: **Supplementary Figure 6**. Expression of THY1 and MSC surface markers in the individual subset from OA and RA. The MFI of THY1 (A) and other MSC surface markers (B) was evaluated with flow cytometry. Data was shown in each subset from OA and RA. OA and RA samples are plotted as white circles and white squares, respectively. Data were analyzed by Holm-Sidak’s multiple comparison test for comparing each subset from the same samples (*p<0.05, **p<0.01, ***p<0.001, ****p<0.0001).

**Additional file 7**: **Supplementary Figure 7**. Osteogenic potentials of SF subsets from OA and RA. A. Quantification of the staining. Cells per donor were cultured in 3 dishes and the results of 10 donors (OA:7, RA:3) for alizarin red and 11 donors for ALP were plotted (OA:10, RA:1). B. Gene expression levels by qPCR. The expression levels of the genes were plotted as a ratio to the expression level of 18S of undifferentiated SF as a control. Cells per donor were cultured in 3 dishes and the results of 10 donors (OA:9, RA:1) for *RUNX2* and *ALPL*, and 9 donors (OA:8, RA:1) for *OCN*. OA and RA samples are plotted as white circles and white squares, respectively. The average data of each subset are plotted as black squares. Data were analyzed by Dunn’s multiple comparison test for comparing each subset from the same patients (*p<0.05, **p<0.01).

**Additional file 8**: **Supplementary Figure 8**. Chondrogenic potentials of SF subsets from OA and RA. A. Histology of the chondrocyte pellets. Chondrocyte matrix was stained as red by safranin O staining and measured the ratio of stained area (A). Cells per donor were cultured in 3 pellets and the results of 18 donors (OA: 10, RA:8). B. qPCR of chondrogenic differentiation marker. The relative expression levels of *ACAN* in differentiated cells. Cells per donor were cultured in 3 pellets and the results of 12 donors (OA:6, RA:6). The target genes were normalized with 18S of undifferentiated SF as a control. OA and RA samples are plotted as white circles and white squares, respectively. The average data of each subset are plotted as black squares. Data were analyzed by Dunn’s multiple comparison test for comparing each subset from the same samples (*p<0.05).

**Additional file 9**: **Supplementary Figure 9.** Adipogenic differentiation markers of SF subsets from OA and RA. Cells per donor were cultured in 3 dishes and the results of 7 donors (OA:3, RA:4). The target genes were normalized with 18S of undifferentiated SF as a control. OA and RA samples are plotted as white circles and white squares, respectively. The average data of each subset are plotted as black squares. Data were analyzed by Dunn’s multiple comparison test for comparing each subset from the same samples.

## Data Availability

The datasets generated during and analyzed during the current study are available from the corresponding author on reasonable request. The microarray data used in the current study is available in https://www.ncbi.nlm.nih.gov/geo/geo2r/?acc=GSE109450&platform=GPL18573.

## Abbreviations

SF: Synovial fibroblast
RA: Rheumatoid arthritis
OA: Osteoarthritis
MSCs: Mesenchymal stem cells
b/c DMARD: Biologic/conventional disease-modified anti-rheumatic drug
MFI: Mean fluorescence intensity
qPCR: Quantitative polymerase chain reaction
IL: Interleukin
TNF: Tumor necrosis factor
MTX: Methotrexate
ALP: Alkaline phosphatase
OCN: Osteocalcin
ACAN: Aggrecan
LPL: Lipoprotein lipase
PPARγ: Peroxisome proliferator-activated receptor γ
siRNA: Small interfering RNA
WST-8: Water-soluble tetrazolium salt
