# Correction: CD34^+^THY1^+^ synovial fibroblast subset in arthritic joints has high osteoblastic and chondrogenic potentials in vitro

**DOI:** 10.1186/s13075-022-02910-x

**Published:** 2022-09-24

**Authors:** Seiji Noda, Tadashi Hosoya, Yoji Komiya, Yasuhiro Tagawa, Kentaro Endo, Keiichiro Komori, Hideyuki Koga, Yasuhiro Takahara, Kazutaka Sugimoto, Ichiro Sekiya, Tetsuya Saito, Fumitaka Mizoguchi, Shinsuke Yasuda

**Affiliations:** 1 Department of Rheumatology, Graduate School of Medical and Dental Sciences, Tokyo Medical and Dental University, 1-5-45, Yushima, Bunkyo-ku, Tokyo, 113-8519 Japan; 2 Center for Stem Cell and Regenerative Medicine, Tokyo Medical and Dental University, Tokyo, Japan; 3 Department of Joint Surgery and Sports Medicine, Graduate School of Medical and Dental Sciences, Tokyo Medical and Dental University, Tokyo, Japan; 4 Department of Orthopedics, Nippon Koukan Fukuyama Hospital, Fukuyama, Japan; 5 Department of Orthopedics, Sonodakai Joint Replacement Center Hospital, Tokyo, Japan

**Correction: Arthritis Res Ther 24, 45 (2022)**

**https://doi.org/10.1186/s13075-022-02736-7**

Following publication of the original article [1], the authors identified typographical errors in PPARγ and CD29 primer sequences in Table 2. The correct table is given below.

**Table 2:** Primers sequences used in the study

Primer	Forward	Reverse
ALPL	5′ATGCTGAGTGACACAGACAAGAAG	5′GGTAGTTGTTGTGAGCATAGTCCAC
RUNX2	5’CATCACCGATGTGCCTAGG	5’TAAGTAAAGGTGGCTGGATAGTG
OCN	5’ GACTGTGACGAGTTGGCTG	5’ GGGAAGAGGAAAGAAGGGTG
ACAN	5’ TGTGGGACTGAAGTTCTTGG	5’AGCGAGTTGTCATGGTCTG
LPL	5’ACACTTGCCACCTCATTCC	5’ ACCCAACTCTCATACATTCCTG
PPARγ	5’GTCGGTTTCAGAAATGCCTTG	5’ GCTGGTCGATATCACTGGAG
THY1	5’ CTACTTATCCGCCTTCACTAGC	5’ TGATGCCCTCACACTTGAC
CD34	5’ CAACATCTCCCACTAAACCCT	5’ TCTTAAACTCCGCACAGCTG
CD73	5’ CACACGGATGAAATGTTCTGG	5’ GGTCAAATGTGCCTCCAAAG
CD271	5’ GTGGAGAGTCTGTGCAGTG	5’ ATCGGTTGTCGGAATGTGG
CD54	5’ CAATGTGCTATTCAAACTGCCC	5’ CAGCGTAGGGTAAGGTTCTTG
CD29	5’ TGTAAGGAGAAGGATGTTGACG	5’ CAACCACACCAGCTACAATTG
18S	5′ACTCAACACGGGAAACCTCA	5′AACCAGACAAATCGCTCCAC

*ALPL* alkaline phosphatase, *OCN* osteocalcin, ACAN aggrecan, LPL lipoprotein lipase, *PPARγ* peroxisome proliferator-activated receptor γ
