# Supplementary figures and images for: CD34+THY1+ synovial fibroblast subset in arthritic joints has high osteoblastic and chondrogenic potentials in vitro
Source: Arthritis Res Ther. 2022 Feb 15;24:45. doi: 10.1186/s13075-022-02736-7 (PMC8845288; doi:10.1186/s13075-022-02736-7)

# Supplementary Figure 1

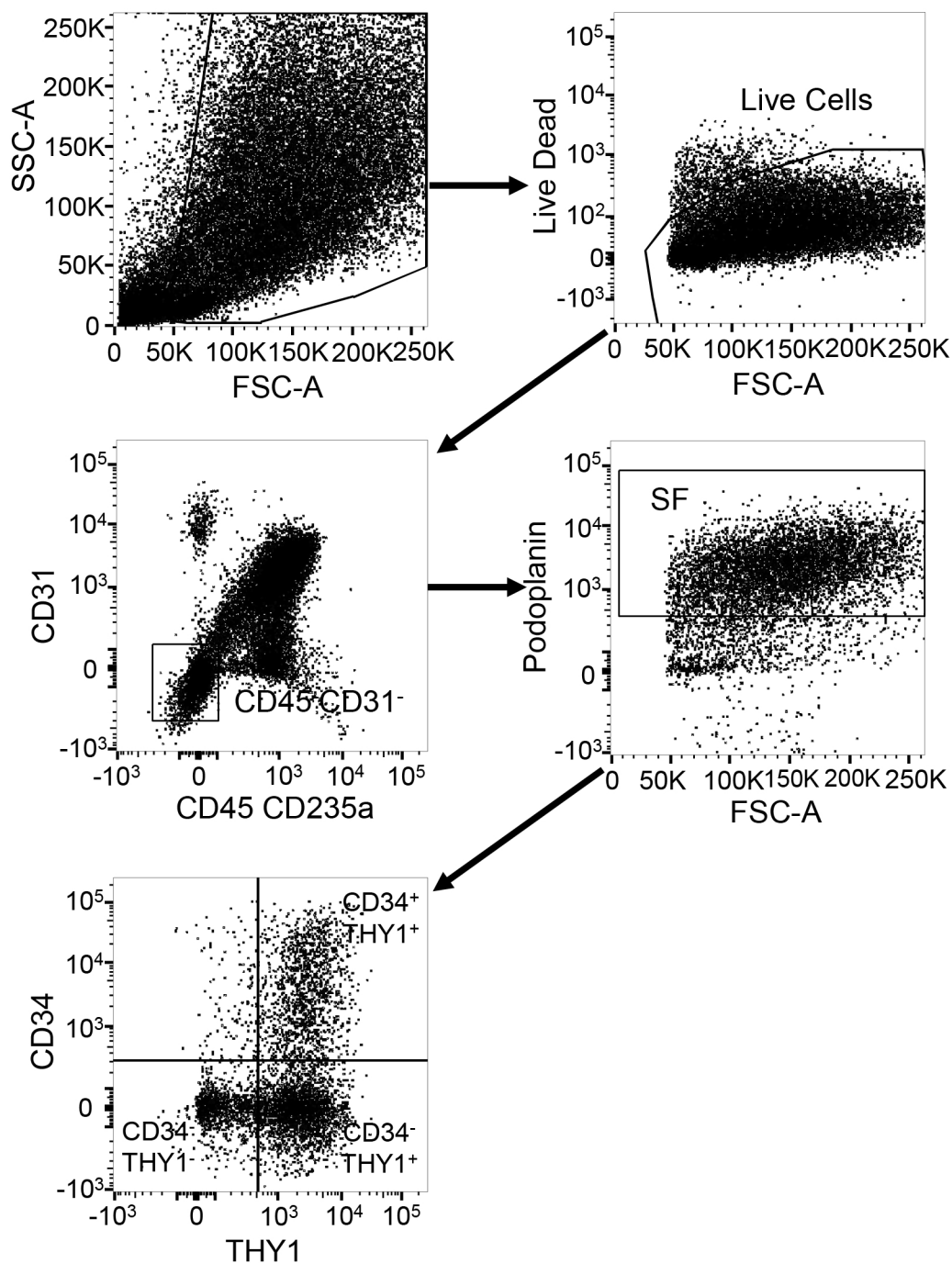

Supplement: Supplementary file 1 — Additional file 1: Supplementary Figure 1. Gating strategy. Gating strategy of freshly isolated cells from OA and RA synovial tissues using flow cytometry. Cells were stained with CD45, CD235a, CD31, THY1 and CD34. Individual subsets were analyzed for the expression of MSC surface markers and were sorted as CD34-THY1-, CD34-THY1+ and CD34+THY1+, respectively. [file 13075_2022_2736_MOESM1_ESM.pdf]

Supplementary Figure 2

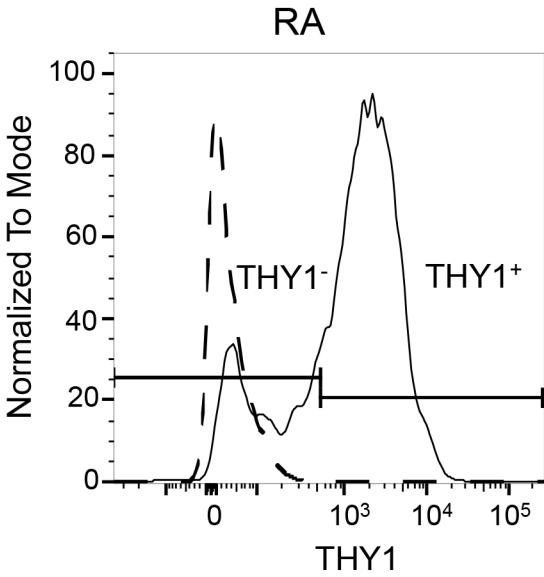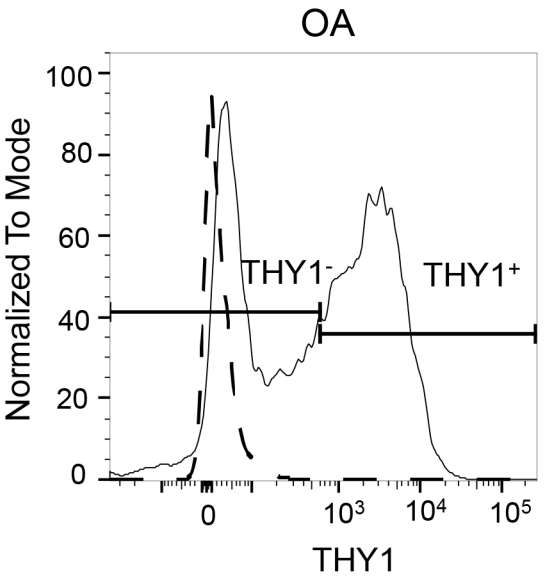

Supplement: Supplementary file 2 — Additional file 2: Supplementary Figure 2. MFI measurement. Positive expression of THY1 and MSC surface markers in SF subsets was identified by comparing fluorescence-minus-one control. THY1 intensity was calculated as MFI of THY1+ SF population. Dash line indicated negative populations, while solid line indicated positive populations. [file 13075_2022_2736_MOESM2_ESM.pdf]

Supplementary Figure 3

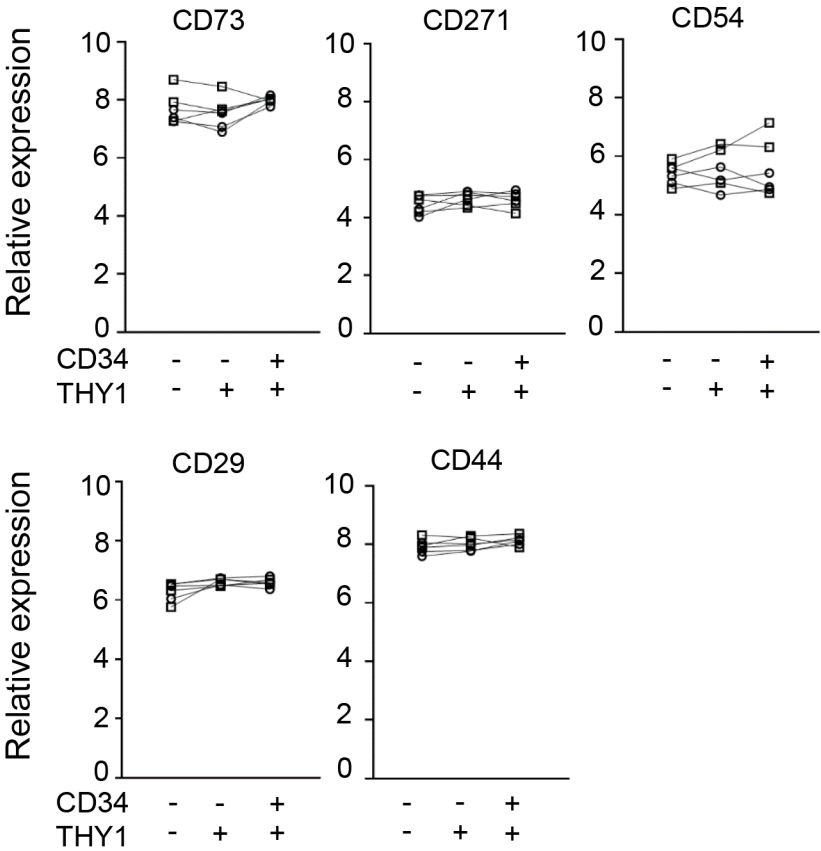

Supplement: Supplementary file 3 — Additional file 3: Supplementary Figure 3. Expression of MSC surface markers. Expression levels of MSC surface markers (CD73, CD271, CD54, CD29 and CD44) on freshly isolated SF subsets were re-analyzed using microarray analysis previously reported (https://www.ncbi.nlm.nih.gov/geo/geo2r/?acc=GSE109450&platform=GPL18573). [file 13075_2022_2736_MOESM3_ESM.pdf]

Supplementary Figure 4

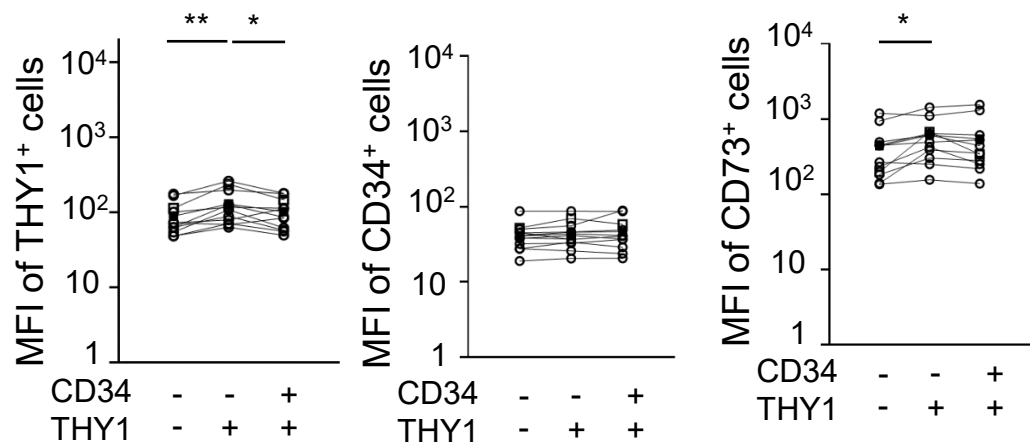

Supplement: Supplementary file 4 — Additional file 4: Supplementary Figure 4. Expression of MSC surface markers after expansion. The MFI of MSC surface markers (THY1, CD34 and CD73) was evaluated with flow cytometry. OA and RA samples are plotted as white circles and white squares, respectively. The average data of each subset are plotted as black squares. Data were analyzed by Holm-Sidak’s multiple comparison test for comparing each subset from the same samples (*p<0.05, **p<0.01). [file 13075_2022_2736_MOESM4_ESM.pdf]

# Supplementary Figure 5

A

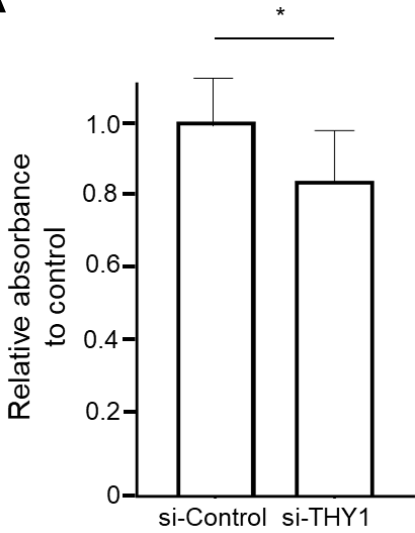

B

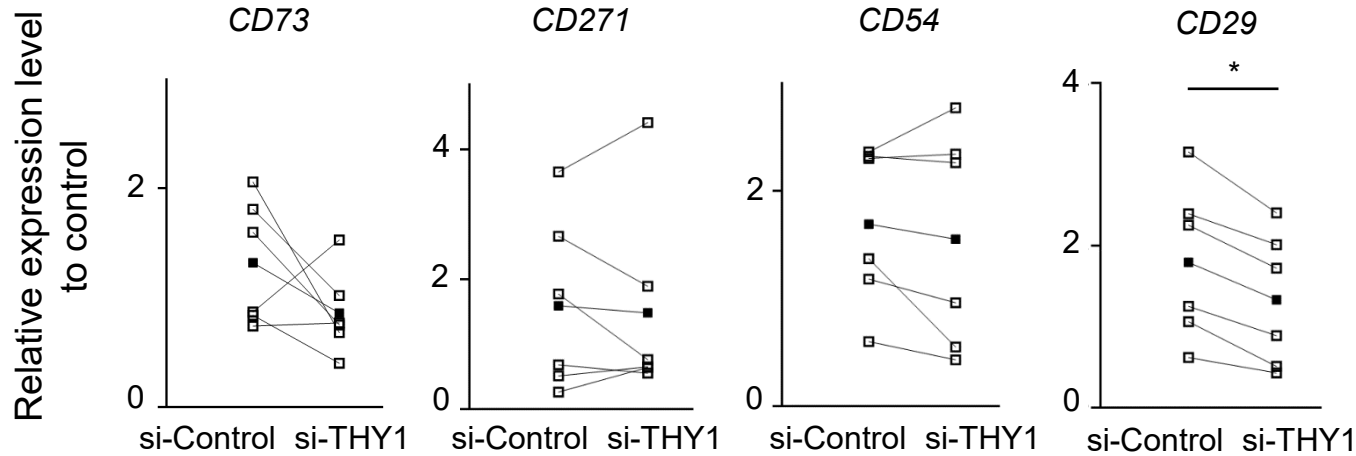

Supplement: Supplementary file 5 — Additional file 5: Supplementary Figure 5. Cell survival/proliferation and MSC surface marker expression by THY1 knockdown. WST-8 assay. Cell survival/proliferation by THY1 knockdown was assessed using WST-8 assay. Data were analyzed by Wilcoxon’s test for comparing each subset from the same samples (*p<0.05). MSC surface marker expression. Expression of MSC surface markers at day 7 after THY1 knockdown was examined by qPCR. The target genes were normalized with 18S of undifferentiated SF as a control. Data were analyzed by Wilcoxon’s test for comparing each subset from the same samples (*p<0.05). [file 13075_2022_2736_MOESM5_ESM.pdf]

Supplementary Figure 6

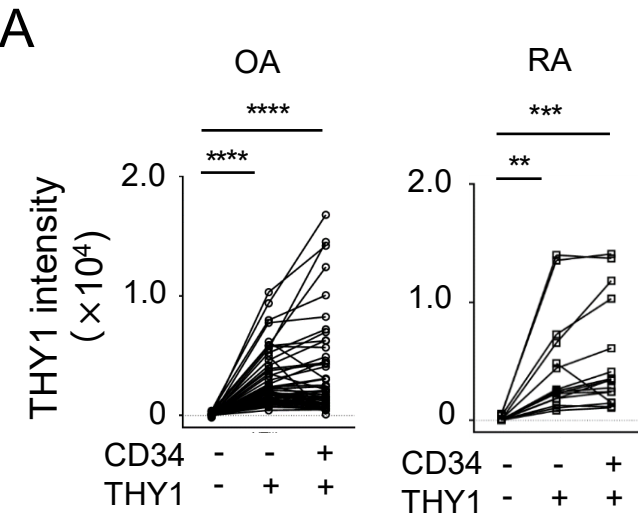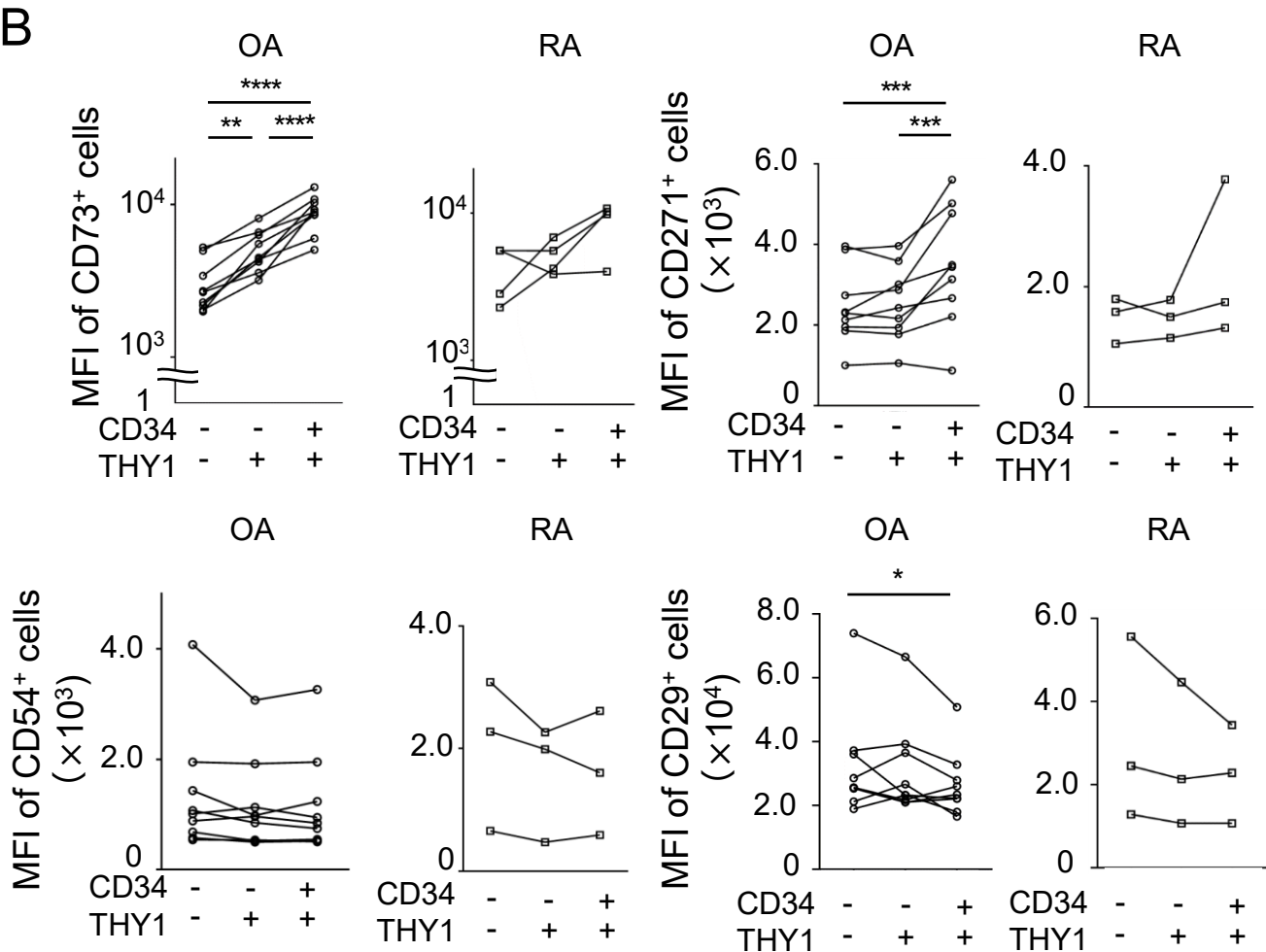

Supplement: Supplementary file 6 — Additional file 6: Supplementary Figure 6. Expression of THY1 and MSC surface markers in the individual subset from OA and RA. The MFI of THY1 (A) and other MSC surface markers (B) was evaluated with flow cytometry. Data was shown in each subset from OA and RA. OA and RA samples are plotted as white circles and white squares, respectively. Data were analyzed by Holm-Sidak’s multiple comparison test for comparing each subset from the same samples (*p<0.05, **p<0.01, ***p<0.001, ****p<0.0001). [file 13075_2022_2736_MOESM6_ESM.pdf]

Supplementary Figure 7

A

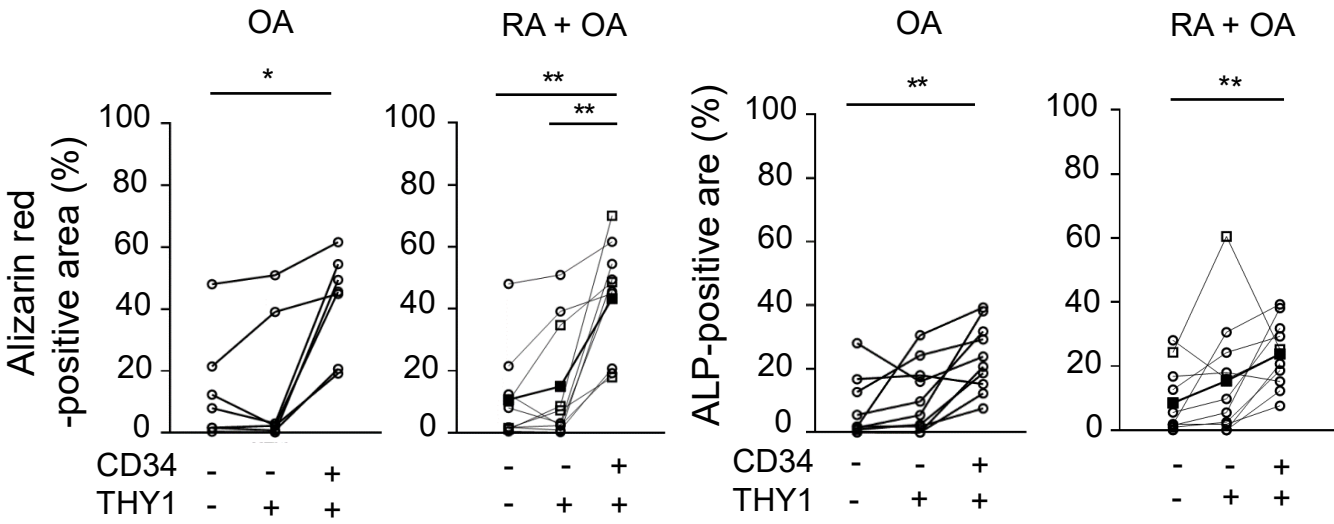

B

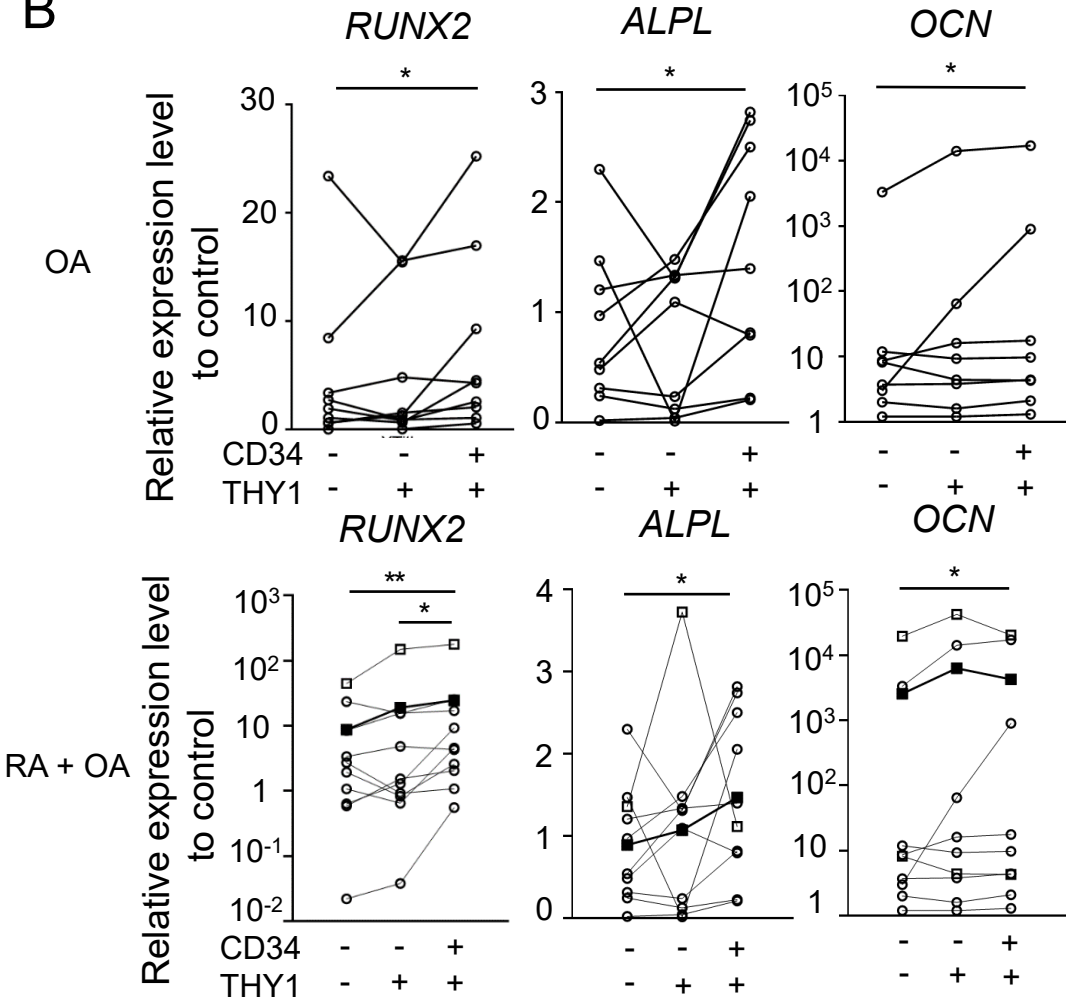

Supplement: Supplementary file 7 — Additional file 7: Supplementary Figure 7. Osteogenic potentials of SF subsets from OA and RA. A. Quantification of the staining. Cells per donor were cultured in 3 dishes and the results of 10 donors (OA:7, RA:3) for alizarin red and 11 donors for ALP were plotted (OA:10, RA:1). B. Gene expression levels by qPCR. The expression levels of the genes were plotted as a ratio to the expression level of 18S of undifferentiated SF as a control. Cells per donor were cultured in 3 dishes and the results of 10 donors (OA:9, RA:1) for RUNX2 and ALPL, and 9 donors (OA:8, RA:1) for OCN. OA and RA samples are plotted as white circles and white squares, respectively. The average data of each subset are plotted as black squares. Data were analyzed by Dunn’s multiple comparison test for comparing each subset from the same patients (*p<0.05, **p<0.01). [file 13075_2022_2736_MOESM7_ESM.pdf]

Supplementary Figure 8

A

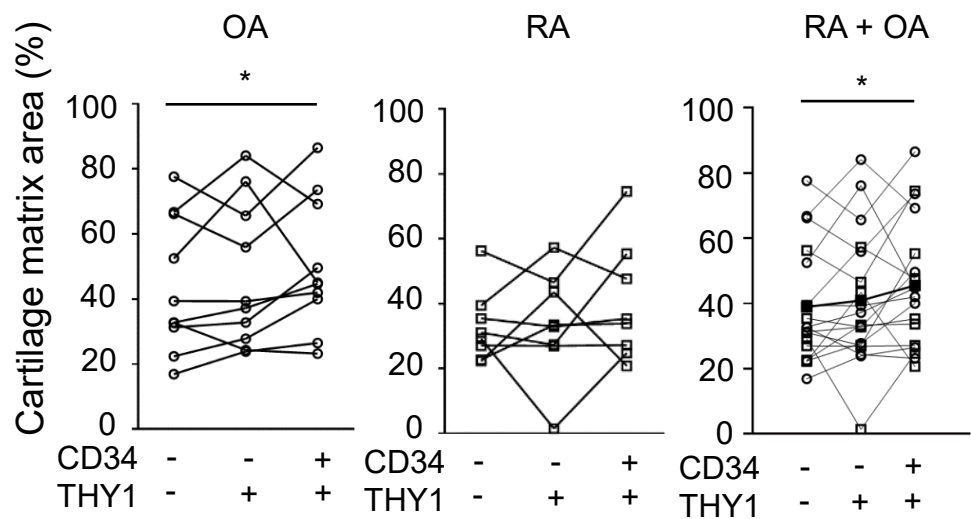

B

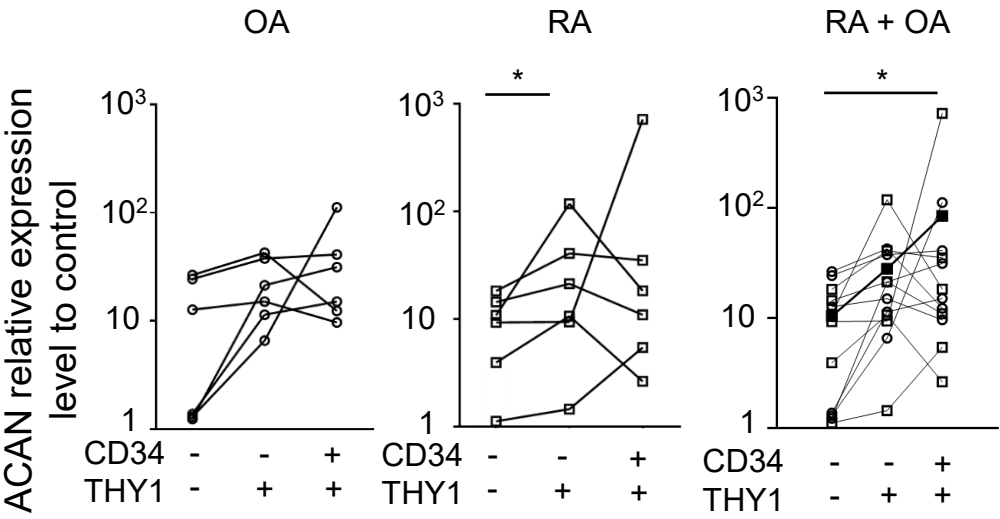

Supplement: Supplementary file 8 — Additional file 8: Supplementary Figure 8. Chondrogenic potentials of SF subsets from OA and RA. A. Histology of the chondrocyte pellets. Chondrocyte matrix was stained as red by safranin O staining and measured the ratio of stained area (A). Cells per donor were cultured in 3 pellets and the results of 18 donors (OA: 10, RA:8). B. qPCR of chondrogenic differentiation marker. The relative expression levels of ACAN in differentiated cells. Cells per donor were cultured in 3 pellets and the results of 12 donors (OA:6, RA:6). The target genes were normalized with 18S of undifferentiated SF as a control. OA and RA samples are plotted as white circles and white squares, respectively. The average data of each subset are plotted as black squares. Data were analyzed by Dunn’s multiple comparison test for comparing each subset from the same samples (*p<0.05). [file 13075_2022_2736_MOESM8_ESM.pdf]

Supplementary Figure 9

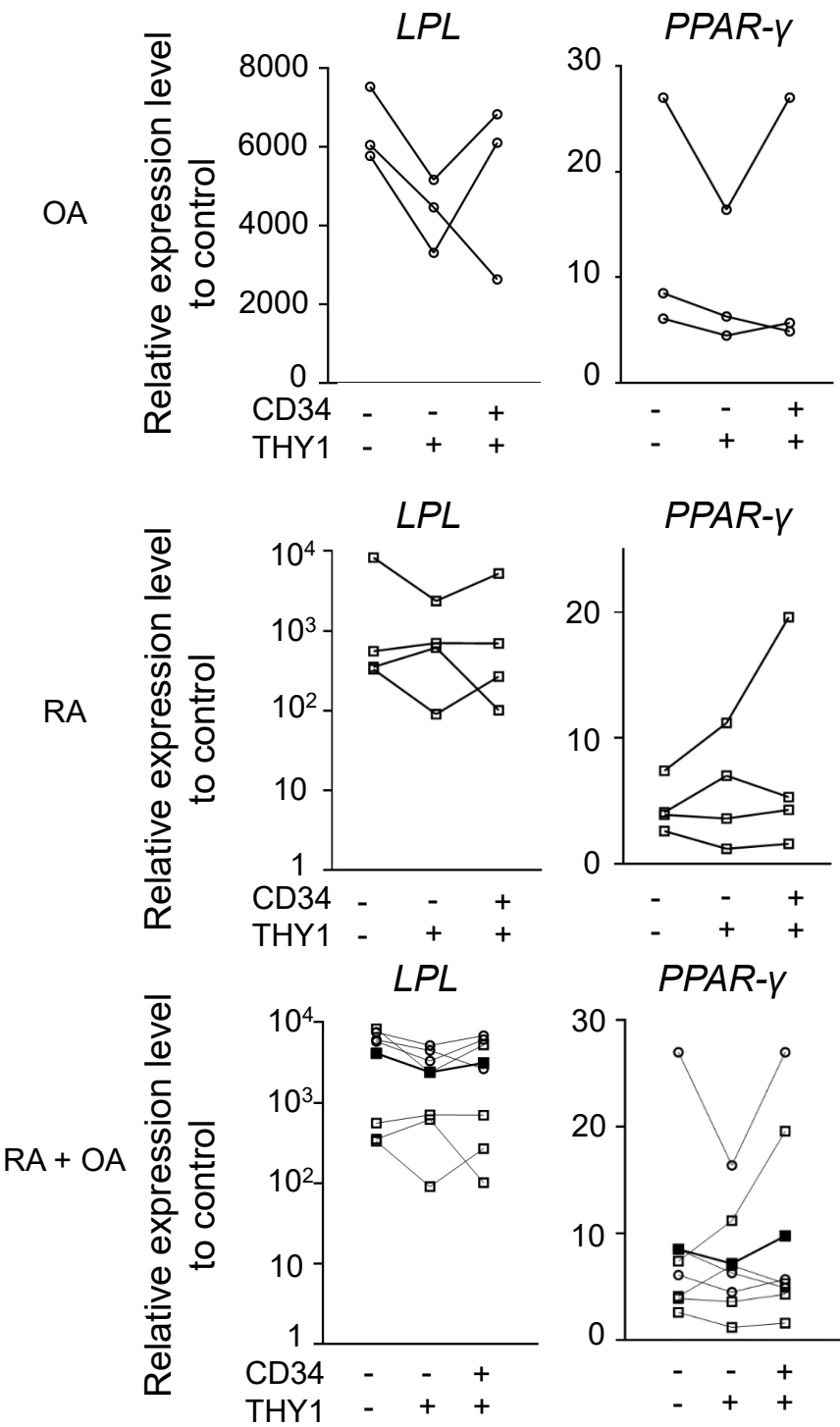

Supplement: Supplementary file 9 — Additional file 9: Supplementary Figure 9. Adipogenic differentiation markers of SF subsets from OA and RA. Cells per donor were cultured in 3 dishes and the results of 7 donors (OA:3, RA:4). The target genes were normalized with 18S of undifferentiated SF as a control. OA and RA samples are plotted as white circles and white squares, respectively. The average data of each subset are plotted as black squares. Data were analyzed by Dunn’s multiple comparison test for comparing each subset from the same samples. [file 13075_2022_2736_MOESM9_ESM.pdf]
